# No evidence for Ago2 translocation from the host erythrocyte into the *Plasmodium* parasite

**DOI:** 10.12688/wellcomeopenres.15852.2

**Published:** 2020-11-20

**Authors:** Franziska Hentzschel, Klara Obrova, Matthias Marti

**Affiliations:** 1 Wellcome Center for Integrative Parasitology; Institute of Infection, Immunity and Inflammation, University of Glasgow, Glasgow, G12 8TA, UK; 2 Center for Infectious Diseases, Parasitology Unit, Heidelberg University Hospital, Heidelberg, 69120, Germany

**Keywords:** Plasmodium, Ago2, host factors

## Abstract

**Background:** *Plasmodium* parasites rely on various host factors to grow and replicate within red blood cells (RBC). While many host proteins are known that mediate parasite adhesion and invasion, few examples of host enzymes co-opted by the parasite during intracellular development have been described. Recent studies suggested that the host protein Argonaute 2 (Ago2), which is involved in RNA interference, can translocate into the parasite and affect its development. Here, we investigated this hypothesis.

**Methods**: We used several different monoclonal antibodies to test for Ago2 localisation in the human malaria parasite, *P. falciparum* and rodent *P. berghei* parasites. In addition, we biochemically fractionated infected red blood cells to localize Ago2. We also quantified parasite growth and sexual commitment in the presence of the Ago2 inhibitor BCI-137.

**Results**: Ago2 localization by fluorescence microscopy produced inconclusive results across the three different antibodies, suggesting cross-reactivity with parasite targets. Biochemical separation of parasite and RBC cytoplasm detected Ago2 only in the RBC cytoplasm and not in the parasite. Inhibition of Ago2 using BCl-137 did not result in altered parasite development.

**Conclusion**: Ago2 localization in infected RBCs by microscopy is confounded by non-specific binding of antibodies. Complementary results using biochemical fractionation and Ago2 detection by western blot did not detect the protein in the parasite cytosol, and growth assays using a specific inhibitor demonstrated that its catalytical activity is not required for parasite development. We therefore conclude that previous data localising Ago2 to parasite ring stages are due to antibody cross reactivity, and that Ago2 is not required for intracellular *Plasmodium* development.

## Background

Malaria, caused by the protozoan parasite *Plasmodium*, remains a devastating disease affecting over 200 million people per year ^1^. Clinical symptoms are initiated when parasites invade and replicate within red blood cells (RBCs). Several potent antimalarials are available, yet drug resistance is spreading as targeted *Plasmodium* proteins and pathways are amenable to mutation ^2^. Host factors required for *Plasmodium* development could potentially offer novel drug targets that are less prone to resistance mutations ^3, 4^. However, while host receptors essential for parasite invasion into the RBC are intensively studied, little is known about host factors required for parasite growth and development within the RBC.

Studies by us and others have suggested possible involvement of the host protein Argonaute 2 (Ago2) in parasite development ^5, 6^. In many eukaryotic systems Ago proteins are core proteins of the RNA interference (RNAi) machinery. Directed by the complementary sequence of microRNAs (miRNAs), Ago proteins bind to target mRNAs and either suppress translation, or, in case of Ago2, the only catalytically active Ago protein in mammalians, directly cleave the mRNA target ^7, 8^. While mature RBCs lack a nucleus and thus *de novo* transcription, Ago2-mediated RNAi is important for RBC maturation from haematopoietic stem cells ^9– 11^, i.e. erythropoiesis. Ago2 is essential for non-canonical processing and maturation of the miRNAs miR-451 and miR-486, major miRNAs involved in erythropoiesis ^12, 13^. Moreover, Ago2 and several miRNAs, including miR-451, are found in *P. falciparum*-infected and uninfected reticulocytes and mature RBCs ^14– 17^. We and others have found previously that infected RBCs (iRBCs) shed increased amounts of extravascular vesicles (EVs) compared to uninfected RBCs ^5, 6^. These iRBC-derived EVs contain Ago2-miRNA complexes that can alter endothelial cell function via RNAi, suggesting a contribution to vascular dysfunction during malaria infection ^5^. Intriguingly, we also localised host Ago2 to the parasite cytoplasm of ring-infected RBCs, suggesting possible Ago2 activity in the parasite ^5, 6^. *Plasmodium* parasites do not have an RNAi machinery including Ago proteins ^18^. To enable manipulation of gene expression via RNAi, we have previously demonstrated successful expression of a human core RNAi machinery in the murine model *Plasmodium berghei* ( *P. berghei*) ^19^. Interestingly, we observed that the ectopic expression of Ago2 in *P. berghei* induced a minor growth defect in asexual parasite stages and downregulation of genes known to be targeted by post-transcriptional repression in female sexual stages (gametocytes). These observations show that Ago2 can interfere with parasite gene expression, supporting the hypothesis that host Ago2 is involved in *Plasmodium* blood stage development. Indeed, a recent study suggested that host Ago2 can downregulate virulence gene expression in *P. falciparum* as part of an innate resistance mechanism of the host RBC against the parasite ^6^.

In this work, we aimed to validate the localisation of host Ago2 to blood stage parasites and investigate if catalytically active host Ago2 is required for parasite development. Our data demonstrate that previously observed Ago2 localisation in the parasite by fluorescence microscopy is the result of antibody cross reactivity. Importantly, we detected no Ago2 in parasites when performing Western blotting on fractionated parasite samples, and inhibition of Ago2 catalytical activity did not impact asexual parasite growth and parasite development. We thus conclude that, at least *in vitro*, Ago2 is not a host factor important for intraerythrocytic parasite development.

## Methods

### Ethics statement

All animal experiments were performed according to European regulations concerning FELASA category B and GVSOLAS standard guidelines. Animal experiments were approved by German authorities (Regierungspraesidium Karlsruhe, Germany), § 8 Abs. 1 Tierschutzgesetz (TierSchG) under the license G-260/12 and were performed according to National and European regulations. Two female outbred NMRI mice (8- to 10-week-old) purchased from Janvier laboratories, France, were used. Mice were kept in groups of 2 to 4 mice per cage under specified pathogen-free (SPF) conditions within the animal facility at Heidelberg University (IBF) on a 12-hour light/dark cycle at 22°C (± 2°C) with *ad libitum* access to food and water.

### *P. falciparum in vitro* culture

Parasite culture was performed as described previously ^20^ *. P. falciparum* parasites (strains 3D7, NF54 and 2004 ^20^) were kept in fresh type 0+ human erythrocytes (NHS National Services Scotland), suspended at 5% hematocrit in HEPES-buffered RPMI 1640 medium (Gibco™ 22400089) supplemented with 10% (w/v) heat inactivated human serum (Interstate Blood Bank), 0.05 mg/ml hypoxanthine (Gibco™) and 50 ng/ml gentamycin (Gibco™). To maintain the plasmid encoding the gametocyte reporter TdTomato, Pf2004 parasites were kept under 4 nM WR99210 (Jacobus Pharmaceuticals) selection pressure. Cultures were kept in a controlled environment at 37°C in a gassed chamber at 5% CO _2_ and 1% O _2_.

### *P. falciparum* gametocyte commitment and mature gametocyte production

Parasite sexual commitment assay was performed as described previously ^20^. Briefly, sorbitol-synchronised *P. falciparum* 2004 parasites expressing TdTomato under the gametocyte promoter etramp10.3 (PF10_0164/PF3D7_1016900) ^20^ were seeded at 24 hpi, 0.5% parasitemia into a 96-well plate adding varying BCI-137 (Merck-Millipore) concentrations (100 µM, 10 µM, 1 µM, 100 nM, 10 nM, 1 nM). For vehicle control, 1 % (v/v) and 0.1% DMSO (corresponding to 100 µM and 10 µM BCI-137, respectively), were added to parasites. Sexual commitment was induced by incubation in LysoPC-depleted minimal-fatty-acid medium for 24 h, while controls were maintained in complete medium. Medium was changed daily maintaining drug pressure. Parasitemia was assessed by SYBR green staining and flow cytometry on day 0, day 2 and day 4 post induction. On day 4, gametocytemia was determined as percentage of TdTomato-positive parasites. Parasite multiplication rate was calculated by dividing parasitemia on day 2 by starting parasitemia on day 0, sexual commitment was calculated by dividing gametocytemia on day 4 by parasitemia on day 2.

For production of mature gametocytes, sexual commitment of the *Pf*NF54 strain was induced as described above. After induction, asexual development was suppressed by addition of heparin to a final concentration of 0.23 mg/ml to the culture medium on days 2, 3 and 4 after induction. Mature gametocytes were obtained after 14 days of culture and fixed and stained for immunofluorescence assays (IFAs) as described below.

### *P. berghei* infections

A female NMRI mouse was infected with *P. berghei* ANKA parasites by intraperitoneal injection of a cryostock containing approximately 1.5 *10^7 infected RBCs in 100 µl blood and 200 µl freezing solution (10% glycerol in alsevier solution). Once parasitemia reached about 2–3%, the mouse was anaesthetised using an overdose of isoflurane and bled by cardiac puncture, and the blood processed for microscopy, as described below.

### IFAs

IFAs were done as described previously ^21^. Mixed-stage cultures of *Pf*3D7 parasites, sorbitol-synchronised *Pf*2004 parasites at 10, 22, 36, and 44 hours post invasion, mature *Pf*NF54 gametocytes or *P. berghei* ANKA parasites were collected and pelleted for 3 min at 1600 rpm. 50 µl RBC pellet were fixed in 1 ml 4% paraformaldehyde (PFA)/0.0075% glutaraldehyde for 20 min at 37°C. Cells were pelleted for 1 min at 3000 rpm and permeabilised in 1 ml 125 mM Glycine/0.1% Triton-X-100 in PBS for 10 min at RT. After pelleting and washing once with 3% BSA/PBS, cells were blocked overnight in 3% BSA/PBS. Primary antibodies were used at a dilution of 1:50 and incubated for 2 h at RT, shaking. RBCs were pelleted and washed 3 times for 10 min with 1% BSA/PBS. Secondary antibodies were diluted 1:300 in 3% BSA/PBS and incubated for 1 h at RT. RBCs were pelleted and washed 3 times for 10 min with PBS. The first washing step included Hoechst in a 1:3000 dilution to stain DNA. Primary and corresponding secondary antibodies used are depicted in Table 1.

**Table 1.** Antibodies used in this study.

Primary Antibody	Secondary antibody (IFA)	Secondary Antibody (WB)
Rabbit-αAgo2 (EPR10411), Abcam Cat# ab186733, RRID:AB_2713978	Goat-αRabbit, Alexa Fluor 488, Thermo Fisher Scientific Cat# A-11008, RRID: AB_143165	IRDye ^®^ 680RD Donkey anti-Rabbit IgG Secondary Antibody, LI-COR Biosciences Cat# 926-68073, RRID:AB_10954442
Mouse-αAgo2 (2E12-1C9), Abcam Cat# ab57113, RRID:AB_2230916	Goat-αMouse, Alexa Fluor 488, Thermo Fisher Scientific Cat# A-11001, RRID: AB_2534069 Goat-αMouse, Alexa Fluor 594, Thermo Fisher Scientific Cat# A-11032, RRID: AB_2534091	IRDye ^®^ 680RD Donkey anti-Mouse IgG Secondary Antibody, LI-COR Biosciences Cat# 926-68072, RRID:AB_10953628
Rat-αAgo2 (11A9), Thermo Fisher Scientific Cat# 14-6519-82, RRID: AB_2784637	Goat-αRat, Alexa Fluor 488, Thermo Fisher Scientific Cat# A-11006, RRID: AB_2534074	**-**
Mouse-α *Pb*Hsp70, RRID:AB_2650482 ^24^	Goat-αMouse, Alexa Fluor 594, Thermo Fisher Scientific Cat# A-11032, RRID: AB_2534091	**-**
Mouse α-αTubulin, Sigma-Aldrich Cat# T5168, RRID:AB_477579	Goat-αMouse, Alexa Fluor 594, Thermo Fisher Scientific Cat# A-11032, RRID: AB_2534091	**-**
Rabbit-α *Pf*BIP (antibody provided by MR4; clone MRA19 ^25^, RRID: AB_2716735)	**-**	IRDye ^®^ 800CW Donkey anti-Rabbit IgG Secondary Antibody, LI-COR Biosciences Cat# 926-32213, RRID:AB_621848
Mouse-αHsHsp70, Santa Cruz Biotechnology Cat# sc-24, RRID: AB_627760	**-**	IRDye ^®^ 800CW Donkey anti-Mouse IgG Secondary Antibody, LI-COR Biosciences Cat# 926-32212, RRID:AB_621847

IFA: Immunofluorescence assay, WB: Western blot.

For imaging, 2 µl of stained parasite pellet were placed onto a glass slide and covered with a coverslip. Imaging was performed on a confocal spinning disc microscope (Nikon) using a 100x objective or a widefield DMi8 microscope (Leica) using a 60x objective and images were processed with FIJI/ImageJ (v 2.0.0-rc-69/1.52p) ^22, 23^.

### Parasite fractionation and western blotting

*P. falciparum Pf*2004 or 3D7 cultures were sorbitol-synchronised and harvested at 10, 22, 36 and 44 hpi and a parasitemia of 5% by pelleting for 3 min at 1600 rpm. For the total sample, 1x10 ^9^ RBCs were washed in 1 ml PBS and resuspended to a final volume of 500 µl parasite lysis buffer supplemented with 1x protease inhibitor cocktail (PIC). For fractionation, 2×10 ^9^ RBCs were washed in 1 ml PBS and lysed for 5 min on ice in 1 ml final volume of 0.015 % saponin/PBS (w/v). After 3 min centrifugation at full speed, the supernatant was transferred to a new tube and supplemented with 1x PIC. The pellet was washed 3 times in 1 ml PBS and resuspended in 50 µl parasite lysis buffer supplemented with 1x PIC.

For western blotting, samples were denatured in 1x SDS loading dye/0.05 M DTT for 5 min at 95°C and 4×10 ^6^ RBCs (total and supernatant) or 2×10 ^7^ iRBC (pellet) per lane were separated on a 4–12% Bis-Tris protein gel (NuPage™) and transferred onto Amersham Hybond LFP 0.2 PVDF Western blotting membranes (GE Life Sciences). Blots were blocked for 1 h in 1% fish gelatin/TBS and primary antibodies ( Table 1, dilution 1:300) incubated over night at 4 °C. Secondary antibodies ( Table 1) were diluted 1:20000 in 1% fish gelatin/0.01% SDS/TBS-T and incubated for 1 h at RT. Blots were developed on a Licor Odyssey ^®^ CLx. Images of the whole blots are depicted in *Extended data*, Extended Figure 1 and 2 ^26^. Raw western blot images are available as *Underlying data* ^27^.

## Results

### Ambiguous Ago localization in parasite and host cell by fluorescence microscopy

**Figure 1.**
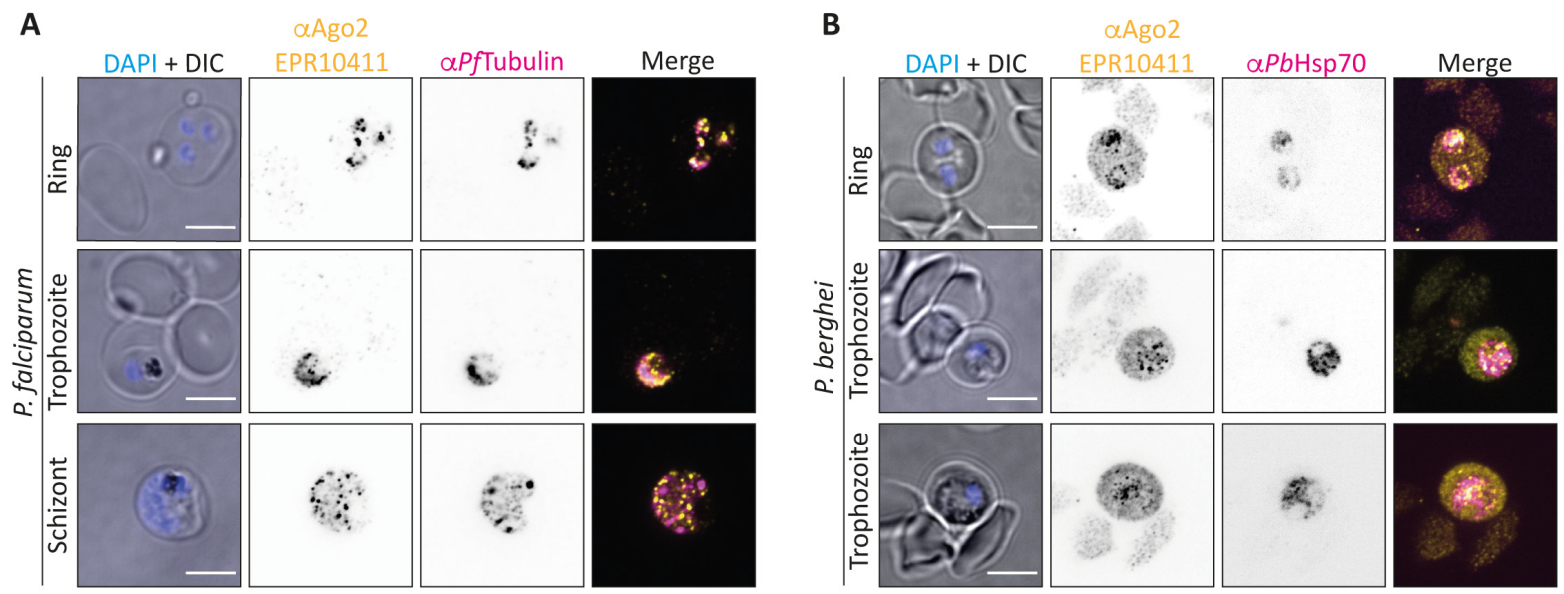
Localisation of Ago2 to the iRBC. ( **A**) IFA of *P. falciparum* 3D7 blood stages. Yellow: Ago2 (EPR10411), magenta: *Pf*Tubulin. ( **B**) IF staining of *P. berghei* ANKA rings and trophozoites. Yellow: Ago2 (EPR10411), magenta: *Pb*Hsp70. Nuclei were stained with Hoechst (blue). Images were taken on a Nikon spinning disc confocal microscope (100x objective). Representative images of at least 5 per condition are shown. Controls using only a secondary antibody revealed no unspecific staining. Scale bar indicates 5 µm.

In a first step, we aimed to verify the previously published results of Ago2 localisation to *P. falciparum* ring stages. To this end, we performed IFAs on mixed *P. falciparum* 3D7 and *P. berghei* ANKA blood stages using the commercial Ago2 antibody EPR10411, which binds to a central peptide epitope of human and murine Ago2. We observed a clear punctuate pattern of host Ago2 in the cytoplasm of all asexual stages of *P. falciparum*, as well as *P. berghei* rings and, to a lesser extent, trophozoites ( Figure 1A, B).

**Figure 2.**
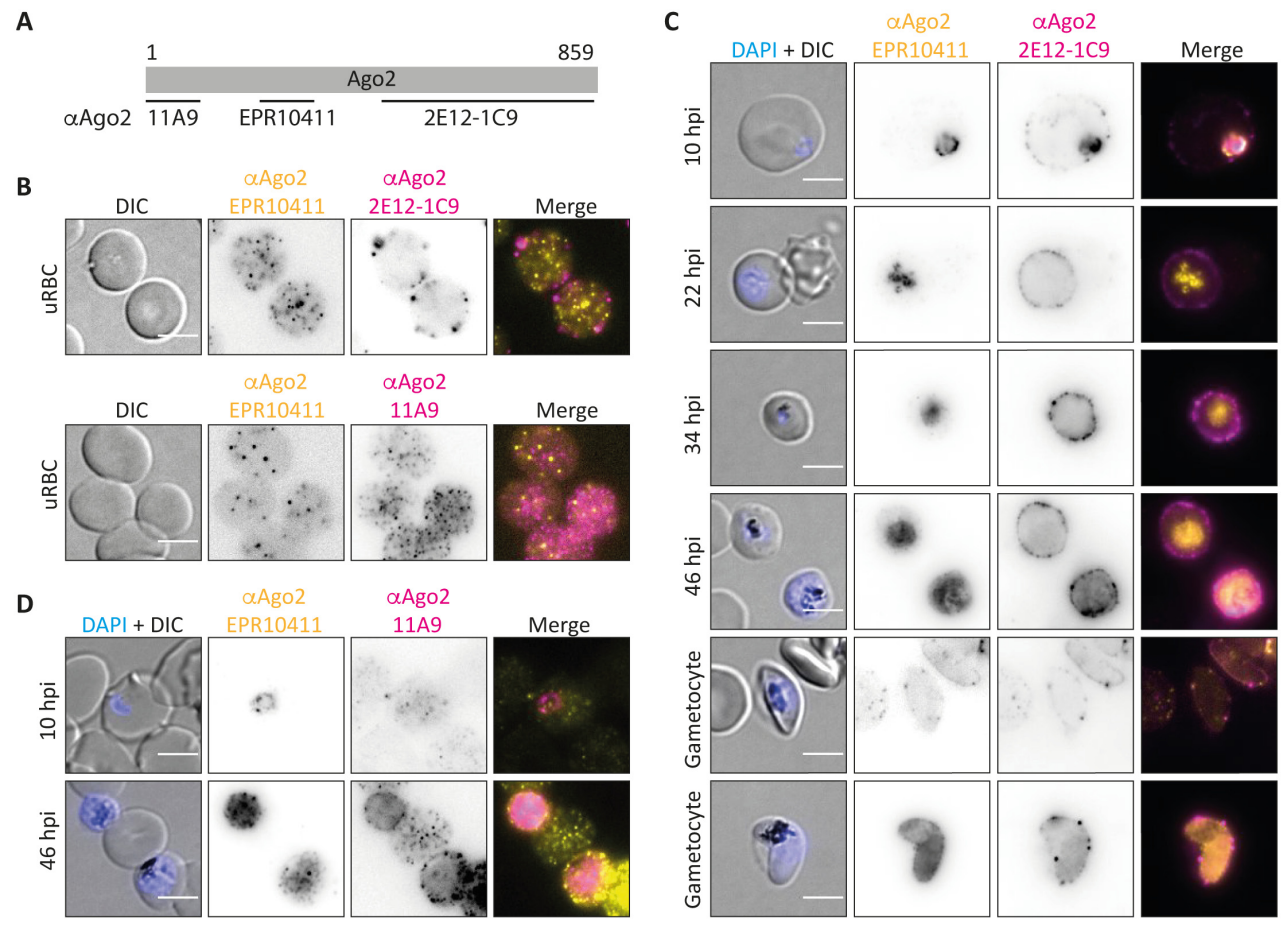
Further analysis of Ago2 localization to the iRBC. ( **A**) Binding sites of αAgo2 antibodies used. The monoclonal antibodies 11A9 and EPR10411 were raised against an N-terminal and a centrally located peptide epitope, respectively. The antibody 2E12-1C9 is a monoclonal antibody raised against the whole C-terminus of hAgo2. ( **B**) IFA of uninfected RBCs. Yellow: Ago2 (EPR10411), magenta: Ago2 (either 2E12-1C9 (top) or 11A9 (bottom)). ( **C**) IFA of *P. falciparum* 2004 parasites at 10, 22, 34 and 46 hours post invasion (hpi) and mature *Pf*NF54 gametocytes. Yellow: Ago2 (EPR10411), magenta: Ago2 (2E12-1C9). Contrast and brightness of EPR10411 panels were individually adjusted to optimise image quality, thus, signal strength cannot be compared between panels. ( **D**) IFA of *P. falciparum* 2004 parasites at 10 and 46 hpi. Yellow: Ago2 (EPR10411), magenta: Ago2 (11A9). Nuclei were stained with Hoechst (blue). Images were taken on a Leica widefield microscope (60x objective). Representative images of at least 5 per condition are shown. Controls using only a secondary antibody revealed no unspecific staining. Scale bar indicates 5 µm.

As this localisation is in contrast to the previous reports which detected Ago2 only in ring stage parasites, we repeated the experiment with two additional commercial monoclonal antibodies (mAbs): 2E12-1C9, which has previously been used ^5, 6^, and 11A9, which has not been tested with *Plasmodium* before. The clone 2E12-1C9 was raised against the Ago2 C-terminus (target epitope unknown), while clone 11A9 was raised against a N-terminal peptide. The respective Ago2 target sites of the different antibodies are depicted in Figure 2A. All three Ago2 antibodies detected Ago2 as punctuate structures in the RBC cytoplasm and close to the membrane, matching the previously published localisation of Ago2 in uninfected RBC ghosts ^17^. However, the antibodies showed only minor overlap in localization, suggesting some non-specific reactivity ( Figure 2B).

To investigate the localization of these antibodies in iRBCs, we used synchronised *Pf*2004 parasites at 10, 22, 36 and 48 hpi, as well as mature gametocytes. Again, only minor overlap in parasite staining was observed across the different antibodies used ( Figure 2C). While EPR10411 labelled all asexual stages (see also Figure 1A) and a fraction of mature *P. falciparum* gametocytes, 2E12-1C9 only stained 10 hpi ring stages, replicating the previously published data with this antibody ^5, 6^. In contrast, 11A9 did not localise to any stage of the parasites ( Figure 2D). In conclusion, three different antibodies directed against Ago2 resulted in three different staining patterns in IFAs strongly suggesting that at least part of the signal is non-specific. Raw images used to generate Figure 1 and Figure 2 are available as *Underlying data* ^27^.

### No evidence for Ago2 localization in the parasite by biochemical fractionation

**Figure 3.**
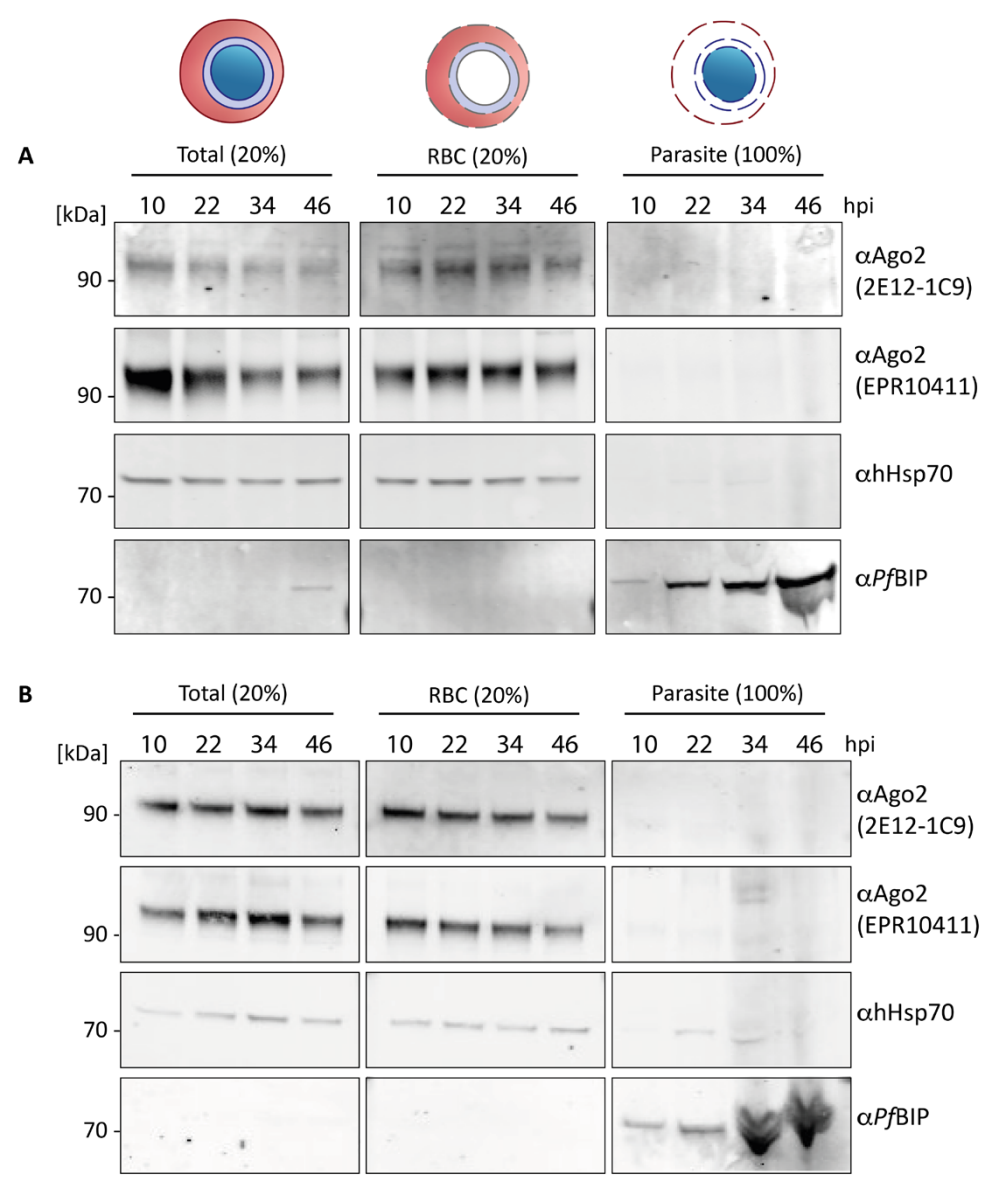
Western blot of fractionated *P. falciparum*-infected iRBCs. **A**) *Pf*2004 or **B**) Pf3D7 iRBCs at 10, 22, 34 and 46 hours post invasion (hpi) were separated via saponin lysis into erythrocyte cytoplasm and PV content (RBC) and parasite cytoplasm and membranes (parasite). Western blot analysis was performed on total protein (left panel, 20 % loaded), saponin supernatant containing RBC and PV cytoplasm (middle panel, 20 % loaded) and pellet containing parasite cytoplasm (right panel, 100 % loaded). Antibodies targeting the RBC housekeeper hHsp70 (expected size human Hsp70: 70 kDa) and the parasite housekeeper *Pf*BIP served as control for proper fractionation. Using two Ago2 antibodies, Ago2 was only detected in the total and the RBC fraction (expected size Ago2 95 kDa).

To independently investigate Ago2 localisation in parasite and host cell, we performed fractionation experiments followed by western blotting. Synchronised *P. falciparum Pf*2004 or *Pf3D7* parasites at 10, 22, 36 and 44 hpi were lysed using saponin and separated into a supernatant fraction containing the RBC cytoplasm and parasitophorous vacuole (PV) content, and the pellet containing membranes and parasite cytoplasm. Western blots of total, supernatant and pellet samples were probed for Ago2 using the antibodies 2E12-1C9 and EPR10411, which both detected Ago2 signal in parasites by IFA ( Figure 1A, Figure 2C). As controls for the fractionation protocol we used antibodies against the RBC cytosol protein human heat shock protein 70 (hHsp70) and the parasite ER protein *Pf*BIP. In non-fractionated (total) samples, we detected bands corresponding to Ago2, hHsp70 and *Pf*BIP ( Figure 3A, B, left panels). As expected, hHsp70 was only detected in the RBC cytosol (RBC, middle panels), while *Pf*BIP was only detected in the parasite (right panels), indicating clean separation of these two fractions. Intriguingly, both antibodies against Ago2 detected a band corresponding to the expected size of Ago2 only in the supernatant fraction (RBC cytosol), independent of parasite stage. No other bands were detected across the whole blots ( *Extended data*, Extended Figures 1, 2A, B) ^26^. This finding contradicts our IFA data ( Figure 1, Figure 2) and suggests that Ago2 is not localized to the parasite.

### Inhibition of Ago2 function does not affect parasite growth

**Figure 4.**
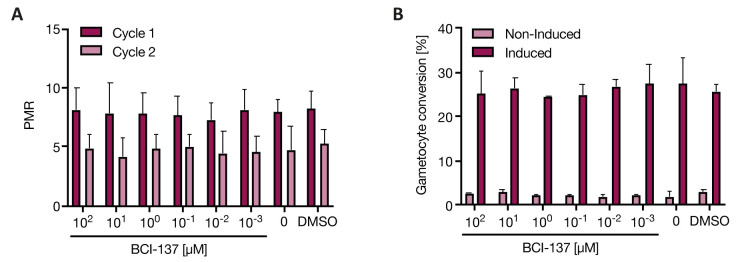
Parasite growth and commitment in presence of an inhibitor of hAgo2. *Pf*2004 parasites were treated with the Ago2 inhibitor BCI-137 at various concentrations or vehicle (DMSO) and assessed for asexual parasitemia and gametocytemia by flow cytometry. **A**) Parasite multiplication rate (PMR) over two cycles of asexual replication. **B**) Gametocyte conversion rate under non-induced and gametocyte-induced conditions. Note: no RBC control is included as it is not possible to directly measure Ago2 activity in RBCs due to their lack of transcriptional activity. However, we used the inhibitor at a tenfold higher maximal concentration compared to what has been shown to be sufficient to inhibit Ago2-miRNA binding ^28^ Shown is the mean ± SD of n = 2, with 3 technical replicates each.

To directly investigate whether host Ago2 plays a role in blood stage malaria parasites, we tested the effect of an Ago2 inhibitor against *P. falciparum* parasites. BCI-137 is a cell permeable Ago2 inhibitor that prevents miRNA-binding and thus functionally inhibits Ago2 ^28^. First, we tested the effect of BCl-137 on parasite growth using a dilution series of the compound. As a previous study inhibiting the host protein acylpeptide hydrolase only observed an inhibitory effect after two asexual replication cycles ^29^, we quantified parasite multiplication rate (PMR) for two consecutive cycles but observed no change in PMR compared to vehicle (DMSO) up to 100 µM of compound ( Figure 4A). We also tested the effect on gametocyte production using our previously published assay to induce sexual commitment via depletion of LysoPC ^20^, and again no phenotype was observed ( Figure 4B). A higher concentration of 1 mM BCI-137 led to a decreased PMR and increased sexual commitment, yet this was due to toxic concentrations of the solvent DMSO ( *Extended data*, Extended Figure 3) ^26^. Flow cytometry output files are available as *Underlying data* ^27^. Altogether these data demonstrate that inhibition of Ago2 function with BCI-137 does not affect parasite multiplication rate or gametocyte production, even at micromolar concentrations.

## Discussion

In this study we have followed up previous reports describing the unusual localisation of a host protein, Ago2, in the intracellular blood stages of *Plasmodium* parasites. Malaria parasites take up nutrients from the host cell environment through nonselective pores in the parasitophorous vacuolar membrane (PVM) ^30, 31^. Large scale uptake of host cytosolic material into the parasite, including haemoglobin and other essential nutrients, is additionally facilitated by a recently characterized endocytosis pathway ^32^. Parasites also take up host enzymes and co-opt their function, as demonstrated by the import of human peroxiredoxin 2 for peroxide detoxification ^33^ or of erythrocyte acylpeptide hydrolase ^29^.

The reported localisation of Ago2 in early ring stage parasites and the observed phenotype upon Ago2 over-expression prompted us to investigate if indeed host Ago2 function had also been co-opted by the malaria parasite. We used three independent approaches to test for a possible role of Ago2 in parasite development. First, we localised Ago2 in iRBCs using a series of monoclonal antibodies that provided ambiguous results. Second, we fractionated iRBCs into host cytoplasm and parasite and performed Western blotting with two antibodies targeting Ago2, demonstrating Ago2 in the RBC fraction only. Third, we measured parasite growth and sexual commitment upon incubation with the Ago2 inhibitor BCI-137, resulting in no discernible phenotype.

In nucleated cells, Ago2 is known to localise both to the cytoplasm and to cytoplasmic ribonucleoprotein granules, e.g. GW/P bodies or stress granules, which are sites of miRNA-mediated mRNA degradation ^34, 35^. To our knowledge, it has not yet been investigated if such granules are present in erythrocytes and the subcellular localisation of Ago2 in RBCs is unknown, with the exception of one study which localised Ago2 to punctuate structures close to the membrane of erythrocyte ghosts; no localisation in the RBC cytoplasm was performed ^17^. In the present work, we observed a speckled localisation for Ago2 within the erythrocyte, resembling the punctuate localisation of Ago2 to P bodies in nucleated cells ^34^. However, in our hands, none of the three different commercial antibodies against Ago2 showed substantial co-localisation in IFA studies. Antibody 2E12-1C9 is known to cross-react with Ago1, Ago3 and Ago4, yet erythroid cells almost exclusively express Ago2 ^12^. Ago2 is subject to a variety of posttranslational modifications, some of which can alter the subcellular localisation of the protein ^36^. For example, phosphorylation of Ago2 at position Ser387 increases its localisation to P bodies ^37^. Intriguingly, the epitope for the antibody EPR10411 encompasses Ser387, and phosphorylation at this site might affect binding of the antibody to Ago2. Other post-translational modifications might exist at the epitopes for the other antibodies, and it is thus tempting to speculate that the different antibodies recognise different subpopulations of post-translationally modified Ago2 in the RBC.

Using three different antibodies, we also observed ambiguous Ago2 staining in the parasite, with the antibody 11A9 not localizing to the parasite at all, the antibody 2E12-1C12 localizing only to ring stages, and the antibody EPR10411 to all asexual parasites and some gametocytes. Importantly, we did not detect Ago2 in the parasite fraction at any time point of the asexual cycle by western blot using two different antibodies, arguing against the presence of Ago2 in the parasite cytoplasm. We previously detected a weak band in the parasite fraction of trophozoites with one of these antibodies ^5^. This may have been the result of incomplete separation of the fractions, as in absence of an antibody targeting a cytoplasmic RBC marker protein, a contamination of the parasite fraction with host cytosol protein cannot be excluded. Similarly, a western blot in another study demonstrated Ago2 in the parasite fraction, but lacked appropriate controls to demonstrate clean fractionation ^6^.

In absence of a *Plasmodium* Ago2 homologue, it remains unknown which parasite protein is detected by αAgo2 antibodies in IFAs. A BLAST search with the sequence of the linear peptide epitope of the antibody EPR10411 (as obtained from the supplier) against a Pf database did not yield any meaningful match. In addition, Western blotting of parasite fractions did not yield any clean band across the whole blot ( *Extended data*, Extended Figures 1, 2) ^26^, indicating that the observed Ago2 localization in the parasite with both antibodies using IFA is the result of cross reactivity with a conformational parasite epitope. In IFAs, EPR10411 produced a specific and strong signal across all parasite stages which increased during asexual development, while 2C12-E2 was only detected in early ring stages, suggesting that the two antibodies cross-react with different parasite antigens.

We also did not observe any inhibitory effect using the membrane-permeable drug BCI-137 on parasite development. This finding is in line with previous observations that ectopic expression of Ago2-dependent miRNAs in *P. berghei* does not regulate gene expression in absence of ectopically expressed Ago2 ^19^. Altogether our data do not support the hypothesis that Ago2 is transferred from host to parasite, and/or that it performs a relevant role in parasite development.

Our study emphasises the importance of using proper controls and orthogonal methods to corroborate initial findings. Notably, the Ago2 antibody 11A9 has been previously reported to cross-react with the nuclear protein SMARCC1 in chromatin immunoprecipitation (IP) studies ^38^. Given that we were not able to detect Ago2 in *Plasmodium*, we presume that previous results of an Ago2-IP from parasites were also influenced by cross reactivity ^6^. In summary, we conclude that there is no Ago2-mediated miRNA activity in malaria parasites, despite a previous report suggesting otherwise.

## Data availability

### Underlying data

Figshare: No evidence for Ago2 translocation from the host erythrocyte to the Plasmodium parasite. https://doi.org/10.6084/m9.figshare.c.49606 ^27^.

This project contains the following underlying data:

- Figure 1B - PbANKA + EPR10411 - Raw Image Files (TIF; https://doi.org/10.6084/m9.figshare.12229037).
- Figure 1B - Pf3D7 + EPR10411 - Raw Image Files (TIF; https://doi.org/10.6084/m9.figshare.12229050). Figure 2C - EPR10411 + 2E12-1C9 - Raw Image Files (TIF; https://doi.org/10.6084/m9.figshare.12229107).
- Figure 2D - Figure 2D - EPR10411 + 11A9 Raw Image Files (TIF; https://doi.org/10.6084/m9.figshare.12229068).
- Figure 3 - Western Blots Raw Data (TIF; https://doi.org/10.6084/m9.figshare.12229169).
- Figure 4, Extended Figure 1 - Flow Cytometry Raw Data (XLSX; https://doi.org/10.6084/m9.figshare.12229160).

### Extended data

Figshare: Extended Figures 1, 2 and 3. https://doi.org/10.6084/m9.figshare.12229028 ^26^.

This project contains the following extended data:

- **Extended Figure 1 (PDF). Whole Western blots of fractionated *P. falciparum*-infected iRBCs (strain Pf2004).** iRBCs at 10, 22, 34 and 46 hours post invasion (hpi) were separated via saponin lysis into into erythrocyte cytoplasm and PV content (RBC) and parasite cytoplasm (parasite). Western blot analysis was performed on total protein (total, 20 % loaded), saponin supernatant containing RBC and PV cytoplasm (RBC, 20 % loaded) and pellet containing parasite cytoplasm (parasite, 100 % loaded). (A) Antibody αAgo2 2E12-1C9 (expected size Ago2 95 kDa). (B) Antibody αAgo2 EPR10411 (expected size Ago2 95 kDa). (C) Antibody αhHsp70 (expected size human Hsp70: 70 kDa). (D) Antibody αPfBIP (expected size 72 kDa). kDa: kilo Dalton, hpi: hours post invasion, M: Marker Li-Cor Chamaeleon DUO.
- **Extended Figure 2 (PDF). Whole Western blots of fractionated *P. falciparum*-infected iRBCs (strain *Pf*3D7).** iRBCs at 10, 22, 34 and 46 hours post invasion (hpi) were separated via saponin lysis into into erythrocyte cytoplasm and PV content (RBC) and parasite cytoplasm (parasite). Western blot analysis was performed on total protein (total, 20 % loaded), saponin supernatant containing RBC and PV cytoplasm (RBC, 20 % loaded) and pellet containing parasite cytoplasm (parasite, 100 % loaded). (A) Antibody αAgo2 2E12-1C9 (expected size Ago2 95 kDa). (B) Antibody αAgo2 EPR10411 (expected size Ago2 95 kDa). (C) Antibody αhHsp70 (expected size human Hsp70: 70 kDa). (D) Antibody αPfBIP (expected size 72 kDa). kDa: kilo Dalton, hpi: hours post invasion, M: Marker Li-Cor Chamaeleon DUO.
- **Extended Figure 3 (PDF). Parasite growth and commitment in presence of an inhibitor of hAgo2.** Pf2004 parasites were treated with the Ago2 inhibitor BCI-137 at various concentrations or vehicle (DMSO) and assessed for asexual parasitemia and gametocytemia by flow cytometry. (A) Parasite multiplication rate (PMR) over two cycles of asexual replication. (B) Gametocyte conversion rate under non-induced and gametocyte-induced conditions. DMSO (1%) is the vehicle control corresponding to 100 μM BCI-137, 0.1 % DMSO 1000 is the vehicle control corresponding to 10 μM BCI-137. Shown is the mean ± SD of n = 2, with 3 technical replicates each.

Data are available under the terms of the Creative Commons Zero "No rights reserved" data waiver (CC0 1.0 Public domain dedication).

## References

[1] World Health Organization: World Malaria Report 2019.2019 Reference Source

[2] Blasco B Leroy D Fidock DA: Antimalarial drug resistance: Linking *Plasmodium falciparum* parasite biology to the clinic. *Nat Med.* 2017;23(8):917–928. 10.1038/nm.4381 28777791 PMC5747363

[3] Langhorne J Duffy PE: Expanding the antimalarial toolkit: Targeting host-parasite interactions. *J Exp Med.* 2016;213(2):143–153. 10.1084/jem.20151677 26834158 PMC4749928

[4] Glennon EKK Dankwa S Smith JD: Opportunities for Host-targeted Therapies for Malaria. *Trends Parasitol.* 2018;34(10):843–860. 10.1016/j.pt.2018.07.011 30122551 PMC6168423

[5] Mantel PY Hjelmqvist D Walch M: Infected erythrocyte-derived extracellular vesicles alter vascular function via regulatory Ago2-miRNA complexes in malaria. *Nat Commun.* 2016;7:12727. 10.1038/ncomms12727 27721445 PMC5062468

[6] Wang Z Xi J Hao X: Red blood cells release microparticles containing human argonaute 2 and miRNAs to target genes of *Plasmodium falciparum*. *Emerg Microbes Infect.* 2017;6(8):e75. 10.1038/emi.2017.63 28831191 PMC5583671

[7] Meister G: Argonaute proteins: functional insights and emerging roles. *Nat Rev Genet.* 2013;14(7):447–459. 10.1038/nrg3462 23732335

[8] Jonas S Izaurralde E: Towards a molecular understanding of microRNA-mediated gene silencing. *Nat Rev Genet.* 2015;16(7):421–433. 10.1038/nrg3965 26077373

[9] Cheloufi S Dos Santos CO Chong MM: A dicer-independent miRNA biogenesis pathway that requires Ago catalysis. *Nature.* 2010;465(7298):584–589. 10.1038/nature09092 20424607 PMC2995450

[10] O’Carroll D Mecklenbrauker I Das PP: A Slicer-independent role for Argonaute 2 in hematopoiesis and the microRNA pathway. *Genes Dev.* 2007;21(16):1999–2004. 10.1101/gad.1565607 17626790 PMC1948855

[11] Listowski MA Heger E Bogusławska DM: MicroRNAs: Fine tuning of erythropoiesis. *Cell Mol Biol Lett.* 2013;18(1):34–46. 10.2478/s11658-012-0038-z 23124859 PMC6276011

[12] Jee D Yang JS Park SM: Dual Strategies for Argonaute2-Mediated Biogenesis of Erythroid miRNAs Underlie Conserved Requirements for Slicing in Mammals Article Dual Strategies for Argonaute2-Mediated Biogenesis of Erythroid miRNAs Underlie Conserved Requirements for Slicing in Mam. *Mol Cell.* 2018;69(2):265–278.e6. 10.1016/j.molcel.2017.12.027 29351846 PMC5824974

[13] Yang JS Lai EC: Dicer-independent, Ago2-mediated microRNA biogenesis in vertebrates. *Cell Cycle.* 2010;9(22):4455–4460. 10.4161/cc.9.22.13958 21088485 PMC3048044

[14] Azzouzi I Moest H Wollscheid B: Deep sequencing and proteomic analysis of the microRNA-induced silencing complex in human red blood cells. *Exp Hematol.* 2015;43(5):382–392. 10.1016/j.exphem.2015.01.007 25681748

[15] Rathjen T Nicol C McConkey G: Analysis of short RNAs in the malaria parasite and its red blood cell host. *FEBS Lett.* 2006;580(22):5185–5188. 10.1016/j.febslet.2006.08.063 16963026

[16] Xue X Zhang Q Huang Y: No miRNA were found in Plasmodium and the ones identified in erythrocytes could not be correlated with infection. *Malar J.* 2008;7:47. 10.1186/1475-2875-7-47 18328111 PMC2329658

[17] Basu A Harper S Pesciotta EN: Proteome analysis of the triton-insoluble erythrocyte membrane skeleton. *J Proteomics.* 2015;128:298–305. 10.1016/j.jprot.2015.08.004 26271157 PMC4619114

[18] Baum J Papenfuss AT Mair GR: Molecular genetics and comparative genomics reveal RNAi is not functional in malaria parasites. *Nucleic Acids Res.* 2009;37(11): 3788–3798. 10.1093/nar/gkp239 19380379 PMC2699523

[19] Hentzschel F Mitesser V Fraschka SA: Gene knockdown in malaria parasites via non-canonical RNAi. *Nucleic Acids Res.* 2020;48(1):e2. 10.1093/nar/gkz927 31680162 PMC7145648

[20] Brancucci NMB Goldowitz I Buchholz K: An assay to probe *Plasmodium falciparum* growth, transmission stage formation and early gametocyte development. *Nat Protoc.* 2015;10(8):1131–1142. 10.1038/nprot.2015.072 26134953 PMC4581880

[21] Tonkin CJ van Dooren GG Spurck TP: Localization of organellar proteins in *Plasmodium falciparum* using a novel set of transfection vectors and a new immunofluorescence fixation method. *Mol Biochem Parasitol.* 2004;137(1):13–21. 10.1016/j.molbiopara.2004.05.009 15279947

[22] Schindelin J Arganda-Carreras I Frise E: Fiji: an open-source platform for biological-image analysis. *Nat Methods.* 2012;9(7):676–682. 10.1038/nmeth.2019 22743772 PMC3855844

[23] Schneider CA Rasband WS Eliceiri KW: NIH Image to ImageJ: 25 years of image analysis. *Nat Methods.* 2012;9(7):671–675. 10.1038/nmeth.2089 22930834 PMC5554542

[24] Tsuji M Mattei D Nussenzweig RS: Demonstration of heat-shock protein 70 in the sporozoite stage of malaria parasites. *Parasitol Res.* 1994;80(1):16–21. 10.1007/BF00932618 8153120

[25] Mantel PY Hoang AN Goldowitz I: Malaria-infected erythrocyte-derived microvesicles mediate cellular communication within the parasite population and with the host immune system. *Cell Host Microbe.* 2013;13(5):521–534. 10.1016/j.chom.2013.04.009 23684304 PMC3687518

[26] Hentzschel F Marti M Obrová K: Extended Figures 1 and 2. *figshare.*Figure.2020 10.6084/m9.figshare.12229028.v1

[27] Hentzschel F Marti M Obrová K: No evidence for Ago2 translocation from the host erythrocyte to the *Plasmodium* parasite. *figshare.*Collection.2020 10.6084/m9.figshare.c.4960649.v1 PMC7808052 33501380

[28] Masciarelli S Quaranta R Iosue I: A small-molecule targeting the microRNA binding domain of argonaute 2 improves the retinoic acid differentiation response of the acute promyelocytic leukemia cell line NB4. *ACS Chem Biol.* 2014;9(8):1674–1679. 10.1021/cb500286b 24914804

[29] Elahi R Dapper C Klemba M: Internalization of Erythrocyte Acylpeptide Hydrolase Is Required for Asexual Replication of *Plasmodium falciparum*. *mSphere.* 2019;4(3):e00077-19. 10.1128/mSphere.00077-19 31068431 PMC6506615

[30] Desai SA Krogstad DJ McCleskey EW: A nutrient-permeable channel on the intraerythrocytic malaria parasite. *Nature.* 1993;362(6421):643–646. 10.1038/362643a0 7681937

[31] Mesén-Ramírez P Bergmann B Tran TT: EXP1 is critical for nutrient uptake across the parasitophorous vacuole membrane of malaria parasites. *PLoS Biol.* 2019;17(9):e3000473. 10.1371/journal.pbio.3000473 31568532 PMC6786648

[32] Birnbaum J Scharf S Schmidt S: A Kelch13-defined endocytosis pathway mediates artemisinin resistance in malaria parasites. *Science.* 2020;367(6473):51–59. 10.1126/science.aax4735 31896710

[33] Koncarevic S Rohrbach P Deponte M: The malarial parasite *Plasmodium falciparum* imports the human protein peroxiredoxin 2 for peroxide detoxification. *Proc Natl Acad Sci U S A.* 2009;106(32):13323–8. 10.1073/pnas.0905387106 19666612 PMC2726359

[34] Leung AKL Sharp PA: Quantifying Argonaute Proteins In and out of GW/P-bodies: implications in microRNA activities. *Adv Exp Med Biol.*In: *Ten Years of Progress in GW/P Body Research*. (Springer, New York, NY).2013;768:165–182. 10.1007/978-1-4614-5107-5_10 23224970 PMC3893129

[35] Leung AKL: The Whereabouts of microRNA Actions: Cytoplasm and Beyond. *Trends Cell Biol.* 2015;25(10):601–610. 10.1016/j.tcb.2015.07.005 26410406 PMC4610250

[36] Jee D Lai EC: Alteration of miRNA activity via context-specific modifications of Argonaute proteins. *Trends Cell Biol.* 2014;24(9):546–553. 10.1016/j.tcb.2014.04.008 24865524 PMC4149831

[37] Zeng Y Sankala H Zhang X: Phosphorylation of Argonaute 2 at serine-387 facilitates its localization to processing bodies. *Biochem J.* 2008;413(3):429–436. 10.1042/BJ20080599 18476811

[38] Van Eijl RAP Van Den Brand T Nguyen LN: Reactivity of human AGO2 monoclonal antibody 11A9 with the SWI/SNF complex: A case study for rigorously defining antibody selectivity. *Sci Rep.* 2017;7(1):7278. 10.1038/s41598-017-07539-4 28779093 PMC5544689

