# Supplementary material for: No evidence for Ago2 translocation from the host erythrocyte into the Plasmodium parasite
Source: Wellcome Open Res. 2020 Nov 20;5:92. Originally published 2020 May 12. [Version 2] doi: 10.12688/wellcomeopenres.15852.2 (PMC7808052; doi:10.12688/wellcomeopenres.15852.2)
Supplement: Peer review history

# Peer review: Reviewer response for version 2

**DOI:** 10.21956/wellcomeopenres.18055.r41546

**Published:** 2021-01-13

**Authors:** Tim W. Gilberger

**Affiliations:** 1 Department of Biology, University of Hamburg, Hamburg, Germany; 2 Bernhard Nocht Institute for Tropical Medicine, Hamburg, Germany

The authors addressed all my comments and I approve the revised version!

Is the work clearly and accurately presented and does it cite the current literature?

Yes

If applicable, is the statistical analysis and its interpretation appropriate?

Not applicable

Are all the source data underlying the results available to ensure full reproducibility?

Yes

Is the study design appropriate and is the work technically sound?

Yes

Are the conclusions drawn adequately supported by the results?

Partly

Are sufficient details of methods and analysis provided to allow replication by others?

Yes

Reviewer Expertise:

Plasmodium falciparum cell biology

I confirm that I have read this submission and believe that I have an appropriate level of expertise to confirm that it is of an acceptable scientific standard.

# Peer review: Reviewer response for version 2

**DOI:** 10.21956/wellcomeopenres.18055.r41547

**Published:** 2020-12-04

**Authors:** Julian Rayner

**Affiliations:** 1 Cambridge Institute for Medical Research, University of Cambridge, Cambridge, UK

The repetition of fractionation with the same strain as used for the IFA rules out any (always likely limited) chance of strain-specific differences, and the additional details further clarify the finding. No further comments.

Is the work clearly and accurately presented and does it cite the current literature?

Yes

If applicable, is the statistical analysis and its interpretation appropriate?

Not applicable

Are all the source data underlying the results available to ensure full reproducibility?

Yes

Is the study design appropriate and is the work technically sound?

Partly

Are the conclusions drawn adequately supported by the results?

Partly

Are sufficient details of methods and analysis provided to allow replication by others?

Yes

Reviewer Expertise:

Malaria parasite cell biology.

I confirm that I have read this submission and believe that I have an appropriate level of expertise to confirm that it is of an acceptable scientific standard.

# Peer review: Reviewer response for version 1

**DOI:** 10.21956/wellcomeopenres.17389.r39405

**Published:** 2020-07-16

**Authors:** Tim W. Gilberger

**Affiliations:** 1 Department of Biology, University of Hamburg, Hamburg, Germany; 2 Bernhard Nocht Institute for Tropical Medicine, Hamburg, Germany

The manuscript by Hentzschel *et al.* revisited the localization and putative function of human Ago2 in Plasmodium-infected erythrocytes. Recent studies, including a paper published in 2016 by the authors, suggested that hAgo2 can translocate into the parasite and affect its development. This prior work was partially based on IFA using a commercial monoclonal.

 Now the authors re-analyzed this localization by a comparative approach using several different monoclonal antibodies in *P. falciparum *and *P. berghei* infected erythrocytes and probed the function of hAgo2 in parasite maturation using the Ago2 inhibitor BCI-137.

 They show ambiguous localization patterns in IFAs within the parasite that point towards cross-reactivity of the anti-Ago2 antibodies and does not support uptake of hAgo2 into the parasite. This finding was further supported by cell fractionation of parasite-infected erythrocytes that showed Ago2 only in the cytoplasm of the erythrocytes and not in the parasite. Additionally, the BCl-137-based Ago2 inhibition over a wide concentration range did not result in altered parasite development.

 In my view, this work is an important contribution to the field because it sets the record straight using complementary techniques. It also highlights the challenges of using IFAs and the need for a very thorough validation of the antibodies used for protein localization. 

 I have only a few additional comments on this well performed study:

  

- In their original study, the authors used 3D7 and show the staining of ring stage parasites by the monoclonal. In Fig. 2 C they provide their comparative localization study with Pf2004 parasites. Although I very much doubt that there are any strain specific differences, for the sake of a direct comparison, 3D7 based localization would be beneficial. Side note: I could not find a reference for the Pf2004 parasite strain.
- Ago2 inhibition (Fig. 4) – Granting that the authors are using BCl-137 in wide molar range, without a positive control for BCL-137-based inhibition of Ago2, this data point remains debatable.

Is the work clearly and accurately presented and does it cite the current literature?

Yes

If applicable, is the statistical analysis and its interpretation appropriate?

Not applicable

Are all the source data underlying the results available to ensure full reproducibility?

Yes

Is the study design appropriate and is the work technically sound?

Yes

Are the conclusions drawn adequately supported by the results?

Partly

Are sufficient details of methods and analysis provided to allow replication by others?

Yes

Reviewer Expertise:

Plasmodium falciparum cell biology

I confirm that I have read this submission and believe that I have an appropriate level of expertise to confirm that it is of an acceptable scientific standard, however I have significant reservations, as outlined above.

# Peer review: Reviewer response for version 1

**DOI:** 10.21956/wellcomeopenres.17389.r38705

**Published:** 2020-06-01

**Authors:** Julian Rayner

**Affiliations:** 1 Cambridge Institute for Medical Research, University of Cambridge, Cambridge, UK

This study expands on previous work by both the lead authors and others that identified a potential role for human Argonaute2 (Ago2), a protein involved in RNAi, in *Plasmodium falciparum *intraerythrocytic development. Two previous manuscripts established the presence of Ago2 in extracellular vesicles (EVs) released from *P. falciparum *infected erythrocytes, where it could complex with microRNA and impact endothelial cell function. Both studies also reported the presence of Ago2 in *P. falciparum *parasites, rather than solely in the erythrocyte cytosol and released EVs, and some of this previous data implied that Ago2 might play a direct role in regulating *P. falciparum *development. In this study the authors compared multiple different commercial anti-Ago2 antibodies in both immunofluorescence and immunoblot experiments and the results suggested that the previous evidence for Ago2 localisation in *P. falciparum *parasites was due to antibody cross-reaction and incomplete fractionation. An Ago2 inhibitor had no impact on parasite development, and the authors therefore argue that there is no conclusive evidence for Ago2 activity in *P. falciparum *parasites.

 While this kind of study is perhaps unglamorous, it is an important addition to the scientific literature as it clarifies previous data, including data previously published by the authors themselves, and might prevent others pursuing hypotheses that are unlikely to be fruitful. It's indexing is therefore strongly supported. The experiments are generally clear and compelling, and the inclusion of full-length blots as extended figures are an example of publishing best practise that should be commended. While the overall case is clear, some minor details and controls would perhaps complete the story and completely show that the earlier indications of Ago2 functioning within *P. falciparum *parasites were misleading.

1. The authors show that three different anti-Ago2 antibodies result in different, non-overlapping, staining patterns in *P. falciparum *infected erythrocytes, compelling evidence that they are recognising additional, non-Ago2, proteins at the dilutions used for immunofluorescence. The comparability of these data with the previous publications is clearly key. While one of the three antibodies, 2E12-1C9, is the same monoclonal used in both previous studies, each study used 2E12-1C9 from different suppliers. To rule out any possible confounding explanation for the different results (such as antibody mix up from certain suppliers), is it not possible to source exactly the same antibodies used in previous studies and compare them?
2. Both previous studies used the *P. falciparum *3D7 strain, but the key figures in this work used a different strain, Pf2004. While it would seem unlikely that Ago2 would play a strain-specific role in parasite development, for completeness sake it would be useful to rule it out, for example by repeating the fractionation data in Figure 3 (which comprehensively argues against the presence of Ago2 in *P. falciparum *parasites) with 3D7.
3. As noted above, Figure 3 is the most convincing data to rule out Ago2 in the parasite, but the methodology used differs subtly from the author’s previously published fractionation (ref 5), which used sequential tetanolysin then saponin fractionation. That approach indicated Ago2 was found in the parasite at the 24 hour time point only, and was found in the parasitophorous vacuole throughout parasite development. The authors argue that their previous data indicating Ago2 in the parasite was the result of incomplete fractionation, but what about the presence of Ago2 in the parasitophorous vacuole - is this still possible?
4. Given the clear evidence of cross-reactivity of anti-Ago2 antibodies with a *P. falciparum *protein(s), and the presence of a few faint parasite bands recognised by anti-Ago2 antibodies in the extended Figures, is there any indication what these cross-reacting proteins might be? If you BLAST a Pf database with just the segments of Ago2 used to raise antibodies, does anything come up at a convincing level of homology?
5. The BCI-137 inhibition data is clearly negative, but lacks a positive control to show that the concentrations used of this inhibitor from this supplier are active, for example by showing enzymatic inhibition, or inhibition of some other non- *Plasmodium *cell.

Is the work clearly and accurately presented and does it cite the current literature?

Yes

If applicable, is the statistical analysis and its interpretation appropriate?

Not applicable

Are all the source data underlying the results available to ensure full reproducibility?

Yes

Is the study design appropriate and is the work technically sound?

Partly

Are the conclusions drawn adequately supported by the results?

Partly

Are sufficient details of methods and analysis provided to allow replication by others?

Yes

Reviewer Expertise:

Malaria parasite cell biology.

I confirm that I have read this submission and believe that I have an appropriate level of expertise to confirm that it is of an acceptable scientific standard, however I have significant reservations, as outlined above.
